# A box on the river: The phylogenetics and phylogeography of *Eucalyptus baueriana* (*Eucalyptus* sect. *Adnataria* ser. *Heterophloiae*)

**DOI:** 10.1371/journal.pone.0276117

**Published:** 2022-11-17

**Authors:** Patrick S. Fahey, Frank Udovicic, David J. Cantrill, Michael J. Bayly

**Affiliations:** 1 School of BioSciences, The University of Melbourne, Parkville, Victoria, Australia; 2 Royal Botanic Gardens Victoria, South Yarra, Victoria, Australia; Guangxi University, CHINA

## Abstract

We present a phylogeographic study of the tree species *Eucalyptus baueriana* Schauer, which occurs in disjunct areas on the near coastal plains and ranges of the south-east Australian mainland. DArTseq data are used to build a phylogeny including *E*. *baueriana* and closely related taxa to test its monophyly, test the genetic distinctness of the three subspecies of *E*. *baueriana*, and investigate relationships between its disjunct populations. Additionally, we use population structure analysis to investigate the genetic distinctness of populations, and MaxEnt to investigate the environmental factors potentially influencing the species’ distribution. We show *E*. *baueriana* is monophyletic and most closely related to three other Blue Box eucalypt species: *E*. *conica* H.Deane & Maiden, *E*. *dalveenica* T.L.Collins, R.L.Andrew & J.J.Bruhl and *E*. *magnificata* L.A.S.Johnson & K.D.Hill, with some evidence for genetic introgression between these taxa. Within *E*. *baueriana*, the deepest genetic breaks do not correspond with the subspecies classification as the two geographically restricted subspecies, together with samples of the more widespread *E*. *baueriana* subsp. *baueriana* from west of the Gippsland lowlands, form a south-western clade with that is sister to other populations of subsp. *baueriana*. The oldest genetic break in the species occurs in far eastern Gippsland (Victoria), corresponding to one of the shortest geographic disjunctions in the species’ distribution. Genetic breaks in other species have been observed in this region which is broadly referred to as the southern transition zone. Both total annual rainfall and the seasonality of this rainfall are hypothesised to affect the species’ distribution; gaps in its distribution are in areas of higher rainfall that support closed forest and in regions with more winter dominated rainfall.

## Introduction

The eastern coast of the Australian mainland stretches from the continent’s southernmost point at Wilsons Promontory in Victoria to the northernmost point on Cape York Peninsula in North Queensland, crossing a latitudinal gradient of more than 3500 km. This gradient covers climatic regions from tropical in the north to cool temperate in the south [1], leading to dramatic changes in ecosystems and vegetation along the coast [2]. Previous work has identified many transition zones and biogeographical barriers that help to shape the diversity of these changes [2–5].

The Southern Transition Zone (STZ) is one such transition zone and corresponds to areas of lower elevation plains that separate the upland areas of the Great Dividing Range (GDR) and the lowland coastal areas in south-east New South Wales [2, 4]. Additionally, the STZ sits near the boundary of the hot summer temperate and warm summer temperate climatic zones [1]. While many species have been shown to have a genetic break in this region, these breaks are often not concordantly located across species. Several barriers to closed forest taxa have been identified in this region (e.g. the Illawarra district, a low-lying coastal plain [2] and the Shoalhaven River [4]). Patterns in sclerophyllous open forest and woodland species have been less obvious in the region and minimal genetic structuring has been observed in many plant taxa that form part of these communities including *Xanthorrhoea* spp. [6], *Correa* spp. [7] and *Callitris rhomboidea* R.Br. ex A.Rich. & Rich [8]. Different patterns have been observed in *Hardenbergia violacea* (Schneev.) Stearn, which shows high haplotype diversity on the south coast of NSW and evidence of more recent expansion southward from this region [9].

**Fig 1:**
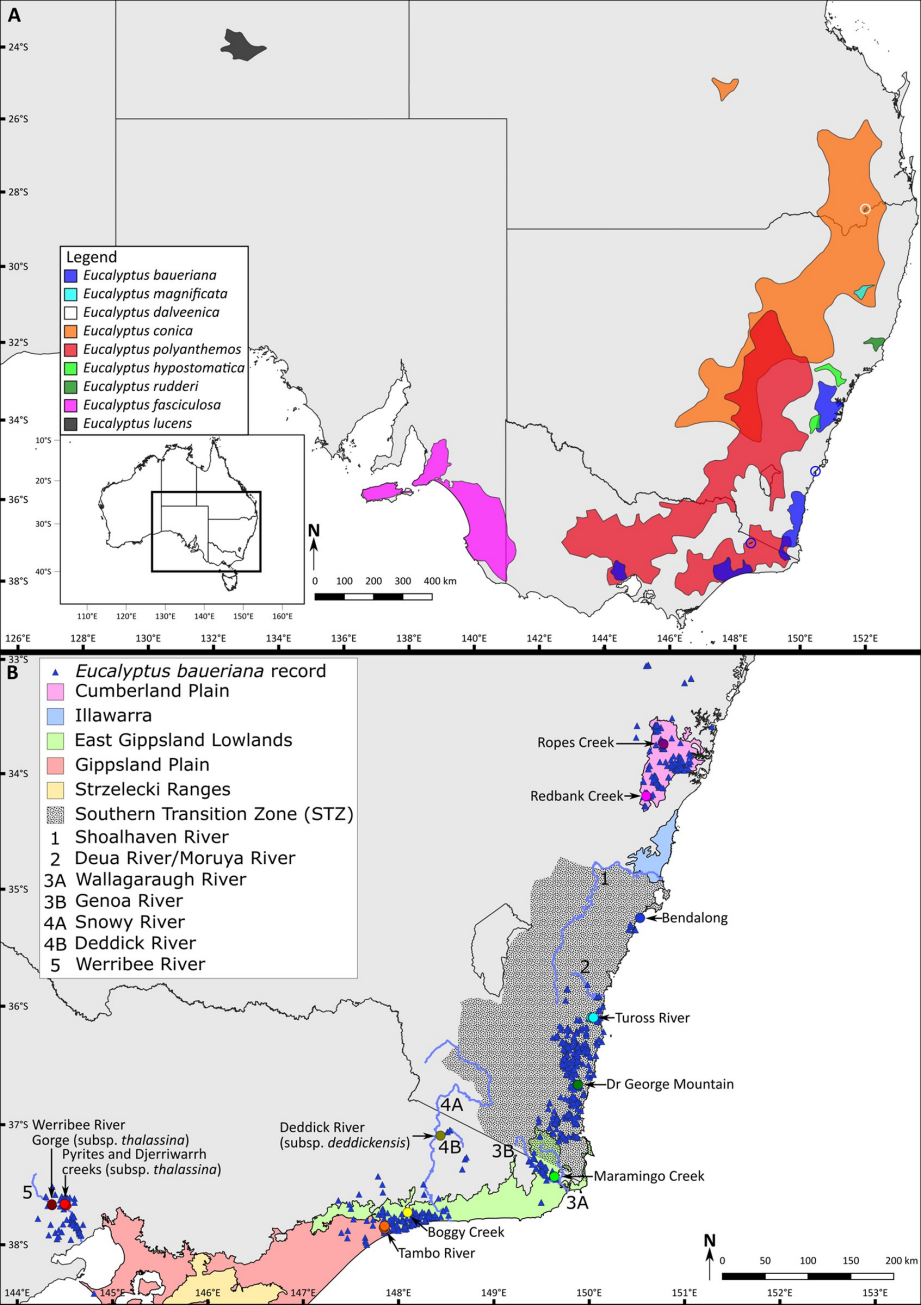
Maps of south-eastern Australia based on records from the Atlas of Living Australia (accessed 30 November 2020) showing (A) distribution of the members of *Eucalyptus* sect. *Adnataria* including *E*. *baueriana*, and (B) the distribution of *E*. *baueriana* including locations of samples used in this study. Bioregions, rivers and the Southern Transition Zone (STZ) discussed in the text are shown in panel B. Bioregions are those of the Interim Biogeographic Regionalisation for Australia Version 7 [12] subregion classification (adapted from [13]). While the boundaries of the STZ are not defined, we have based our understanding of it on the thoughts of previous authors [2, 4]. *Eucalyptus baueriana* records downloaded from the Atlas of Living Australia are indicated with blue triangles and points representing collections of the species are coloured and labelled to match Figs 2 and 3. The base map is adapted from [14] under a CC BY 3.0 AU license.

In this study we set out to further elucidate patterns of genetic diversity in sclerophyllous plant taxa of the STZ by investigating patterns of genetic diversity in *Eucalyptus baueriana* Schauer, Blue Box, a member of *Eucalyptus* section *Adnataria* series *Heterophloiae* (Fig 1A). *Eucalyptus* ser. *Heterophloiae* contains nine species that predominately occur in woodlands and open forests in south-eastern Australia (Fig 1A). The only species to occur outside this area, the mallee (multi-stemmed) species *E*. *lucens* Brooker & Dunlop that occurs on arid slopes in rocky ranges west of Alice Springs in central Australia [10] (Fig 1A), has been suggested to be the most distinct member of the complex [11]. *Eucalyptus fasciculosa* F.Muell. is also geographically distinct from all other members of the complex, occurring on Kangaroo Island and much of south-eastern South Australia from the Mount Lofty Ranges south-east across the Victorian border (Fig 1A). A pair of morphologically similar and closely related species, considered by Nicolle [11] to be potentially conspecific, *E*. *rudderi* Maiden and *E*. *hypostomatica* L.A.S.Johnson & K.D.Hill are restricted to central NSW [10]. The former is limited to a single coastal area around Taree while the latter occurs in two distinct areas in the ranges around the Sydney Basin: around Lake Burragorang and in the hills that form the southern border of the lower Hunter Valley (Fig 1A). The southern population of *E*. *hypostomatica* is parapatric with *E*. *baueriana* populations on the Cumberland Plain.

Another species in the group that overlaps in distribution with *E*. *baueriana* is *E*. *polyanthemos* Schauer, which occurs in woodlands on the inland slopes of the GDR from central Victoria to the southern edge of the Brigalow Belt between Dubbo and Goulburn River National Park and on the coastal side of the GDR in Gippsland from the Traralgon region into far south-east NSW [10]. These two species are differentiated where they co-occur by several features including the parts of the landscape they occupy; *E*. *baueriana* is a species of riparian vegetation whereas *E*. *polyanthemos* most commonly occurs on ridges and slopes away from riparian zones. Morphologically, the species are differentiated by their habit and degree of rough bark coverage, with *E*. *baueriana* typically a more robust species that always has rough, box-type bark [10] covering the trunk and large branches, a trait that is sometimes absent in *E*. *polyanthemos*, and by the fruit and leaves, with *E*. *baueriana* having more obconical fruit and glossy green leaves which are noticeably thinner and more delicate than the leathery leaves of *E*. *polyanthemos* [10].

The three remaining species in the series, *E*. *conica* H.Deane & Maiden, *E*. *magnificata* and *E*. *dalveenica* T.L.Collins, R.L.Andrew & J.J.Bruhl, do not have overlapping distributions with *E*. *baueriana* but together with it were termed the Blue Boxes by Collins et al. [14]. *Eucalyptus conica* is widespread on the inland slopes of the GDR and adjacent plains from the West Wyalong-Young-Cowra region north to the south Burnett region and Barakula State Forest in Queensland, with an outlying population to the north-west in and around Carnarvon National Park. Maiden [15] considered this species to be conspecific with *E*. *baueriana*, although this taxonomy has not been followed by any authors since. The distribution of *E*. *conica* completely encompasses that of both *E*. *magnificata*, which is only found on the south-east Armidale Plateau in the New England region of NSW, and the recently described *E*. *dalveenica* known initially from a single, extremely restricted population near the town of Dalveen in the Granite Belt region of southern Queensland [16], although the Stanthorpe Rare Wildflower Consortium has confirmed via email (gbwildflowers@gmail.com, May 24^th^ 2022) that a second population of the species has subsequently been discovered on private land approximately 3 km from the initial population. Prior to the description of *E*. *magnificata* [17], and *E*. *dalveenica* [16], populations of these taxa were included in a broader circumscription of *E*. *baueriana* [18].

In 2011, Rule [19] described two new subspecies of *E*. *baueriana*, both restricted to isolated populations in Victoria. *Eucalyptus baueriana* subsp. *thalassina* includes all populations in the Werribee River catchment west of Melbourne and was considered to be morphologically distinct based upon its “smaller habit, smaller buds with shorter pedicels and smaller fruits” [19]. Populations on the Deddick River in eastern Victoria were classified as *E*. *baueriana* subsp. *deddickensis* Rule and were described as having a smaller stature than *E*. *baueriana* subsp. *baueriana*, along with darker green adult leaves and smaller buds and fruit [19]. Since these publications, these subspecies have not been recognised by many other authors [e.g. 10, 11], but are considered valid by the Australian Plant Census [20].

*Eucalyptus baueriana* occurs in several discrete areas on the south-east coast of Australia between the Sydney Basin and Werribee River catchment west of Melbourne (Fig 1B). The species primarily occurs in fertile soils in riparian corridors along creeks and rivers in open forests and woodlands [19], and it is absent from many river catchments in the south-east corner with higher rainfall that supports closed forest. The westernmost populations occur in the Werribee River catchment west of Melbourne (Fig 1B) and are those described as *E*. *baueriana* subsp. *thalassina* by Rule [11]. The largest remaining population in this catchment occurs along Pyrites and Djerriwarrh creeks in the Long Forest Flora and Fauna Reserve; a biogeographical oddity which supports the only mallee vegetation south of the GDR [21]. These isolated *E*. *baueriana* populations are separated by more than 300 km from the nearest populations to the east on the boundary of the Gippsland plain and the East Gippsland Lowlands (Fig 1B). Much of this disjunction lies across the Gippsland Plain (Fig 1B), which has been hypothesised to be a biogeographical break in closed forest taxa [2]. However, as *E*. *baueriana* is not a closed forest species the environmental factors maintaining this disjunction likely differ from these taxa. North of these populations, several extremely restricted populations along the Deddick River, a tributary of the Snowy River (Fig 1B), represent the second geographically restricted subspecies, *E*. *baueriana* subsp. *deddickensis* Rule [11]. The populations of this subspecies are the most inland known of the species and occur in a landscape of rough, steep terrain with deep river gorges that is very different to the coastal plains and hills typical of the species.

East of the eastern Gippsland Plains/western East Gippsland Lowlands and Deddick River populations, the species inhabits the upper reaches of the Genoa River/Wallagaraugh River catchment, also in the East Gippsland Lowlands region (Fig 1B); with consistent occurrences along many water courses north from here as far as the Deua and Moruya rivers at Moruya. A further restricted population on the south coast of NSW occurs in the Bendalong region just south of the Illawarra district. This population is not associated with a particular watercourse, rather inhabiting a small area of coastal woodland on Bendalong Point. These populations are within the STZ as defined by both Milner et al. [4] and Bryant and Krosch [2]. The species is absent from the Illawarra region and ranges north of there but occurs along several watercourses on the Cumberland Plains in the Sydney Basin. While no previous phylogenetic nor distribution-wide population genetic studies have been undertaken on this species, populations were sampled from the Cumberland Plain and Bendalong Point in a study of *E*. *magnificata* L.A.S.Johnson & K.D.Hill, and these populations were shown to be somewhat genetically and morphologically distinct from one another [16].

In this study, we aim to establish the monophyly of *E*. *baueriana* within *Eucalyptus* series *Heterophloiae* and whether there is any genetic support for the recognition of the subspecies of Rule [19]. We then investigate patterns of genetic relatedness between the disjunct populations of *E*. *baueriana* and build hypotheses regarding the environmental processes that have established and maintained this unique distribution. We do this by developing a reduced representation genetic dataset utilising Diversity Arrays Technologies sequencing (DArTseq) [22, 23] on both samples of all species in *E*. series *Heterophloiae* along with outgroups of various phylogenetic distance, and population level sampling from across the range of *E*. *baueriana*. We present phylogenetic analyses of the complete dataset and a STRUCTURE analysis on our *E*. *baueriana* samples.

## Methods

### Sampling

We had initially aimed to collect ninety total samples of *E*. *baueriana*, ten samples from nine sites across the species distribution. However, due to small population sizes and limits to travel due to COVID-19, actual sample numbers per population ended up ranging from one to ten, and we added two further sampling sites bringing the total number of sites to eleven. As we could only access a single stand of fourteen individuals on a ~150 m stretch of the Deddick River in the Snowy River National Park due to the steep landscape, we limited our sample to the eight most spread-out individuals at the site to limit sampling of siblings, leaving a total of eighty-three samples of the species. All field collections consisted of approximately ten leaves placed into individually labelled coffee filters and then into silica desiccant beads for rapid drying. At least one representative specimen was taken from each collection site for accessioning in the University of Melbourne Herbarium (Table 1). All collecting was undertaken under scientific collecting permits granted by the Victorian Department of Sustainability and Environment (permit number 10008557), New South Wales National Parks and Wildlife Service (permit number SL102100), and South Australian Department of Environment and Water (permit number Q26766-2).

**Table 1:** Collection details for samples used in this study. For *E*. *baueriana* collection sites, where multiple samples were collected, geographic coordinates are the mean of the individual sample coordinates.

Species	Placement in classification of Nicolle [11]	Samples	Representative herbarium specimen	Collection locality	Average coordinates
*E*. *albens*	*E*. sect. *Adnataria* ser. *Subbuxeales*	PSF88	MELUD122655a	Wongarbon Nature Reserve, New South Wales	-32.30312, 148.77977
*E*. *albopurpurea*	*E*. sect. *Adnataria* ser. *Subbuxeales*	CC560	MELUD122661a	American River lookout, Kangaroo Island, South Australia	-35.80631, 137.74009
*E*. *behriana*	*E*. sect. *Adnataria* ser. *Buxeales*	PSF69E	MELUD122643a	Long Forest Flora and Fauna Reserve, Victoria	-37.66377, 144.50092
*E*. *baueriana* subsp. *baueriana*	*E*. sect. *Adnataria* ser. *Heterophloiae*	PSF108A–J	MELUD128413a, MELUD128414a	Tambo River and Metung Road, north of Metung, Victoria	-37.85363, 147.84928
*E*. *baueriana* subsp. *baueriana*	*E*. sect. *Adnataria* ser. *Heterophloiae*	PSF109A–B	-	Boggy Creek, Nowa Nowa, Victoria	-37.73146, 148.09438
*E*. *baueriana* subsp. *baueriana*	*E*. sect. *Adnataria* ser. *Heterophloiae*	PSF112A–J	MELUD128417a	Maramingo Road, Maramingo Creek, Victoria	-37.43287, 149.63282
*E*. *baueriana* subsp. *baueriana*	*E*. sect. *Adnataria* ser. *Heterophloiae*	PSF113A–J	MELUD128418a	Doctor George Mountain Road, south of Brogo River, Tarraganda, New South Wales	-36.66466, 149.88093
*E*. *baueriana* subsp. *baueriana*	*E*. sect. *Adnataria* ser. *Heterophloiae*	PSF114A–J	MELUD128419a	Tuross River and Eurobodalla Road, west of Bodalla, New South Wales	-36.09786, 150.03186
*E*. *baueriana* subsp. *baueriana*	*E*. sect. *Adnataria* ser. *Heterophloiae*	PSF115A–J	MELUD128420a	Waratah Street, Bendalong, New South Wales	-35.24701, 150.53272
*E*. *baueriana* subsp. *baueriana*	*E*. sect. *Adnataria* ser. *Heterophloiae*	PSF116A	MELUD128421a	Redbank Creek, Picton, New South Wales	-34.19408, 150.59797
*E*. *baueriana* subsp. *baueriana*	*E*. sect. *Adnataria* ser. *Heterophloiae*	PSF117A–J	MELUD128423a, MELUD128424a	Ropes Creek Boulevard, St Marys, New South Wales	-33.74127, 150.77865
*E*. *baueriana* subsp. *deddickensis*	*E*. sect. *Adnataria* ser. *Heterophloiae*	PSF120A–H	MELUD128427a, MELUD128428a, MELUD128429a	Deddick River near Fall Creek, Deddick Valley, Victoria	-37.09158, 148.43923
*E*. *baueriana* subsp. *thalassina*	*E*. sect. *Adnataria* ser. *Heterophloiae*	MJB2572A–E, MJB2573A–B, PSF68A–C	MELUD122642a	Pyrites and Djerriwarrh creeks, Long Forest Flora and Fauna Reserve, Victoria	-37.66886, 144.50639
*E*. *baueriana* subsp. *thalassina*	*E*. sect. *Adnataria* ser. *Heterophloiae*	PSF132A, PSF132C	MELUD128439a, MELUD128441a	Werribee River, Werribee Gorge State Park, Victoria	-37.66833, 144.36389
*E*. *cajuputea*	*E*. sect. *Adnataria* ser. *Subbuxeales*	PSF48B	MELUD122613a	Devils Peak, Flinders Ranges, South Australia	-32.4154, 138.0014
*E*. *castrensis*	*E*. sect. *Adnataria* ser. *Subbuxeales*	DNicolle5263	MELUD122664a	Pokolbin, New South Wales	-32.75302, 151.23382
*E*. *conica*	*E*. sect. *Adnataria* ser. *Heterophloiae*	CANB480492	CANB 480492.1	Tenterfield, New South Wales	-29.0661, 151.9867
*E*. *conica*	*E*. sect. *Adnataria* ser. *Heterophloiae*	PSF130A	MELUD128437a	Mel Gibson Park, Warwick, Queensland	-28.23850, 152.02363
*E*. *dalveenica*	*E*. sect. *Adnataria* ser. *Heterophloiae*	PSF129A–B	MELUD128436a	Dalveen, Queensland	-28.49593, 151.96393
*E*. *dumosa*	*E*. sect. *Dumaria*	PSF34A	MELUD122581a	Senior Road, north of Bordertown, South Australia	-36.12536, 140.78767
*E*. *fasciculosa*	*E*. sect. *Adnataria* ser. *Heterophloiae*	PSF36A	MELUD122584a	Para Wirra Conservation Park, South Australia	-34.67973, 138.81891
*E*. *froggattii*	*E*. sect. *Adnataria* ser. *Subbuxeales*	PSF99A	MELUD122673a	Richmond Plains-Wedderburn Road, north-west of Wedderburn, Victoria	-36.39333, 143.56944
*E*. *hypostomatica*	*E*. sect. *Adnataria* ser. *Heterophloiae*	MEL2462403	MEL 2462403A	Nattai National Park, New South Wales	-34.14056, 150.47611
*E*. *largiflorens*	*E*. sect. *Adnataria* ser. *Buxeales*	PSF64	MELUD122636a	River Road, between Antwerp and Tarranyurk, Victoria	-36.05597, 142.41306
*E*. *leptophylla*	*E*. sect. *Bisectae*	PSF22I	-	Wychitella Nature Conservation Reserve, Victoria	-36.34839, 143.62355
*E*. *lucens*	*E*. sect. *Adnataria* ser. *Heterophloiae*	MEL0278390	MEL 0278390A	Mount Razorback, Tjoritja West MacDonnell National Park, Northern Territory	-23.5333, 132.4333
*E*. *leucoxylon*	*E*. sect. *Adnataria* ser. *Melliodorae*	PSF98	MELUD122672a	Wychitella Nature Conservation Reserve, Victoria	-36.36444, 143.61
*E*. *magnificata*	*E*. sect. *Adnataria* ser. *Heterophloiae*	CANB540728	CANB 540728.1	Metz Gorge, east of Armidale, New South Wales	-30.5758, 151.8833
*E*. *magnificata*	*E*. sect. *Adnataria* ser. *Heterophloiae*	CANB875142	CANB 875142.1	Long Point Road, south-east of Hillgrove, New South Wales	-30.6125, 151.9417
*E*. *melliodora*	*E*. sect. *Adnataria* ser. *Melliodorae*	PSF106A	MELUD122698a	Royal Park, Melbourne, Victoria	-37.78963, 144.9557
*E*. *microcarpa*	*E*. sect. *Adnataria* ser. *Subbuxeales*	PSF71D	MELUD122646a	Long Forest Flora and Fauna Reserve, Victoria	-37.66367, 144.50787
*E*. *moluccana*	*E*. sect. *Adnataria* ser. *Subbuxeales*	PSF94	-	Menangle Road, Glen Alpine, New South Wales	-34.08188, 150.77838
*E*. *odorata*	*E*. sect. *Adnataria* ser. *Subbuxeales*	PSF36B	MELUD122585a	Para Wirra Conservation Park, South Australia	-34.68059, 138.81903
*E*. *oleosa*	*E*. sect. *Bisectae*	PSF41B	MELUD122591a	Telowie Gorge National Park, South Australia	-33.03633, 138.10168
*E*. *polyanthemos* subsp. *vestita*	*E*. sect. *Adnataria* ser. *Heterophloiae*	PSF70B	MELUD122645a	Long Forest Flora and Fauna Reserve, Victoria	-37.66137, 144.50594
*E*. *polyanthemos* subsp. *marginalis*	*E*. sect. *Adnataria* ser. *Heterophloiae*	PSF97A	MELUD122671a	Wychitella Nature Conservation Reserve, Victoria	-36.36444, 143.61
*E*. *polyanthemos* subsp. *vestita*	*E*. sect. *Adnataria* ser. *Heterophloiae*	PSF110G	MELUD128415a	Russels Track, Simpsons Creek, Victoria	-37.75292, 148.35956
*E*. *polybractea*	*E*. sect. *Adnataria* ser. *Subbuxeales*	PSF27A	MELUD122575a	Greater Bendigo National Park, Victoria	-36.56786, 144.31169
*E*. *porosa*	*E*. sect. *Adnataria* ser. *Melliodorae*	PSF39	MELUD122588a	Rhynie, South Australia	-34.13232, 138.66748
*E*. *rudderi*	*E*. sect. *Adnataria* ser. *Heterophloiae*	MEL0706391	MEL 0706391A	Kiwarrak State Forest, New South Wales	-32.0, 152.3833
*E*. *sideroxylon*	*E*. sect. *Adnataria* ser. *Melliodorae*	PSF95	MELUD122697a	Royal Park, Melbourne, Victoria	-37.79237, 144.95621
*E*. *tricarpa*	*E*. sect. *Adnataria* ser. *Melliodorae*	PSF107	MELUD122700a	Royal Park, Melbourne, Victoria	-37.78965, 144.94822
*E*. *viridis*	*E*. sect. *Adnataria* ser. *Subbuxeales*	PSF76D	MELUD122649a	Charcoal Tank Reserve, South of West Wyalong, New South Wales	-33.98636, 147.15471
*E*. *viridis*	*E*. sect. *Adnataria* ser. *Subbuxeales*	PSF93B	MELUD122660a	Inglewood-Texas Road, North of Yelarbon State Forest, Queensland	-28.45646, 151.08972
*E*. *wimmerensis*	*E*. sect. *Adnataria* ser. *Subbuxeales*	PSF60A	MELUD122631a	Mallee Dam Bushland Reserve, Victoria	-36.40309, 141.49142
*E*. *woolsiana*	*E*. sect. *Adnataria* ser. *Subbuxeales*	PSF92A	MELUD122659a	Inglewood-Texas Road, north of Yelarbon State Forest, Queensland	-28.47847, 151.09396

We supplemented our sampling of *E*. *baueriana* with sampling of all other members of series *Heterophloiae*. Field collected samples in this group included: two samples of *E*. *polyanthemos* from Victoria, two samples of *E*. *dalveenica* from near the township of Dalveen and a sample of *E*. *conica* from the town of Warwick in southern Queensland. We sampled from six herbarium specimens from the Australian National Herbarium and the National Herbarium of Victoria: two samples of *E*. *magnificata*, and one each of *E*. *rudderi*, *E*. *hypostomatica*, *E*. *conica* and *E*. *lucens*. We were also able to take advantage of a previous DArTseq run to supplement our data and provide outgroups, which included two further samples from *E*. series *Heterophloiae*: a sample each of *E*. *polyanthemos* and *E*. *fasciculosa*, completing our sampling of all species in the series. The additional *E*. sect. *Adnataria* samples from this previous work included single samples of twenty-one species from *E*. ser. *Melliodorae*, *E*. ser. *Buxeales* and *E*. ser. *Subbuxeales*. For outgroups we included one member of the sister section *Dumaria*, and two samples from *E*. section *Bisectae*.

### DNA extraction and DArT-seq

A standard CTAB protocol per McLay [24] modified per Fahey et al. [25] was undertaken on 70–80 mg of dried leaf material per sample. DNA pellets were resuspended in 50 μL of 10 mM Tris HCl pH 7 buffer before extraction quality and concentration were quantified using a Nanodrop 2000 spectrophotometer (ThermoFisher) and Qubit 2.0 fluorometer (Invitrogen). Based upon Qubit estimates of DNA concentrations, samples were standardised to 100 ng DNA/μL and sent to Diversity Arrays Technology Pty Ltd, Canberra for DArTseq data generation [22, 23]. Extractions from one field collection and three herbarium specimens were below the target concentration of 100 ng/μL and were included at the concentration of the extraction, the lowest being ~60 ng/μL. While all field collected samples passed initial quality controls for DArTseq, five of the six herbarium specimens (*E*. *magnificata* CANB540728, *E*. *magnificata* CANB875142, *E*. *rudderi* MEL0706391, *E*. *conica* CANB480492 and *E*. *lucens* MEL0278390) initially failed due to low concentrations and short DNA fragments. These samples were rerun at a higher concentration and, while still producing fewer reads than field collections, four of the five returned usable data, with only the sample of *E*. *lucens* (MEL0278390) not producing sufficient reads to be included in our phylogenetic analyses. All datasets used in this study are available for download from Dryad (https://doi.org/10.5061/dryad.wdbrv15q5 [26]).

### Analyses

As the SNP dataset returned by DArT and used in later analyses was not in a format that could easily be used in rooted phylogenetic analyses and had bias in missing data towards outgroup taxa, a known issue in DArTseq datasets, for phylogenetic analyses we reconstructed loci from the raw sequencing reads using *ipyrad* [27]. These reads were first trimmed to remove adaptor and barcode sequences using CutAdapt [28], rendering them 75 bp in length on average. The reads were treated as single end RAD data and assembled using reference mapping to the eleven chromosomes of the *E*. *grandis* reference genome [29]. This approach was taken to remove plastid and mitochondrial loci from the dataset, as it has been shown that the cpDNA phylogeny of the eucalypts often has discordance with nDNA phylogenies [30, 31], which may have influenced our results had these loci not been removed. Default *ipyrad* settings were changed as follows: maximum low-quality bases = 2, minimum samples per locus = 4, cluster threshold = 0.9, maximum SNPs per locus = 10%, maximum indels per locus = 5 and maximum shared heterozygous sites = 0.25. A maximum likelihood (ML) analysis was performed on the PHYLIP file containing the full data alignment produced by *ipyrad* using *RAxML* [32]. The rapid bootstrap analysis and ML tree search algorithm was used for tree generation under a GTR model with BFGS rate optimisation, and one hundred bootstrapping replicates were performed. A maximum parsimony (MP) analysis was undertaken on the SNP alignment generated by *ipyrad* in PAUP* [33], with ambiguous bases treated as polymorphisms and one hundred bootstrap replications performed. Resulting ML and MP trees were viewed in TreeGraph 2 [34] rooting on the two section *Bisectae* samples (*E*. *oleosa* F.Muell. ex Miq. PSF41B and *E*. *leptophylla* F.Muell. ex Miq. PSF22I), and then compared for incongruence by eye. Samples with unexpected placement in resultant phylogenies were tested for introgression due to hybridisation between taxa using ABBA-BABA tests [35] run in the *ipyrad* toolbox.

To investigate patterns of genetic relatedness and diversity across the sampling sites of *E*. *baueriana*, the original SNP dataset generated by DArT was used to perform STRUCTURE analysis [36, 37] as outgroups are not included in this analysis. Data were filtered in R [38] utilising the *dartR* package [39] to include only eighty-one of the *E*. *baueriana* samples and one SNP per marker with a call rate of ≥ 90% and a reproducibility of 1. We excluded two *E*. *baueriana* samples from this analysis: the sample PSF117D from Ropes Creek that did not form part of the monophyletic *E*. *baueriana* clade in the phylogeny, and the single Redbank Creek sample PSF116A. The latter was removed as it was not supported as closely related to any other samples in the phylogeny, thus representing a lone sample from a unique population, and uneven sampling of populations can lead to incorrect inferences of K in STRUCTURE analyses [40]. Additionally, when we did include it in our analyses, at the optimal value of K it showed relatively even contributions from multiple clusters that corresponded to other populations rather than a unique cluster by itself, which we could not meaningfully interpret. Basic population statistics including observed and expected heterozygosity, *F*_is_ and *F*_st_ values were calculated on these SNPs using the relevant functions with *dartR*.

The filtered dataset was exported from R into a.str formatted file for STRUCTURE analysis. This analysis was undertaken using the StrAuto Python program that facilitates automation and parallelisation of runs [41]. Ten replicate runs for values of K one through ten were completed with a burnin of 100 000 followed by a run of length 500 000, with the upper limit of K being chosen based upon the ten sampling sites represented in the dataset. Once the StrAuto run was completed, the probability and delta K was examined to find the optimal values of K (Table 2). Results from these values of K were then summarised across runs using CLUMPP [42] and plotted using the online implementation of STRUCTURE PlotV2 [43].

**Table 2:** Key statistics used to determine optimal values of K (number of clusters) for structure analysis. All values are derived from ten replicate runs at each K value. LnP(K) is minimised at K = 8, while Delta K is highest at K = 2, reflecting the two values of K shown in Fig 3.

K-value	Mean LnP(K)	Stdev LnP(K)	Delta K
1	195630.31	44.1555	NA
2	179027.69	5.6624	1624.836758
3	171625.61	189.6755	1.881898
4	164580.48	1097.1116	2.50722
5	160286.05	442.103	4.189499
6	157843.81	573.4006	0.855353
7	155892.03	617.3827	0.637287
8	153546.8	580.498	75.772365
9	195187.28	122595.4534	0.092503
10	248168.17	149367.7762	NA

#### Species distribution modelling

MaxEnt [44] was used to build species distribution models to assist in building hypotheses regarding the environmental factors that influence the distribution of *E*. *baueriana*. Thirty initial environmental factors were chosen as potential predictors for these models: the nineteen bioclimatic variables available from WorldClim [45], ten soil characteristics (organic carbon content, clay content, silt content, sand content, nitrogen content, phosphorus content, pH, available water capacity, depth of soil and depth of regolith) available from the Soil and Landscape Grid of Australia measured at a depth of 30–60 cm where relevant [46] and a 1 arc-second digital elevation model of Australia [47]. Bioclimatic and elevation rasters were resampled and aligned to match the resolution of the soil data rasters (3 arc-seconds) using a bilinear (2x2 kernel) resampling scheme, and all predictors were clipped to an area of south-east Australia defined by the following IBRA 7 regions [12] in which the species occurs, or which are within 50 km of a known population of the species: Australian Alps, South East Coastal Plain, South East Corner, South Eastern Highlands, Southern Volcanic Plain, Sydney Basin and Victorian Midlands. Occurrence records of *E*. *baueriana* were downloaded from the Atlas of Living Australia [48: accessed June 2020] and initially filtered manually to remove records that were outliers, likely misidentifications or incorrectly georeferenced, and to only include records with unique coordinates.

The *VariableSelection* function from the *MaxentVariableSelection* package [49] in R [38] was used to test and optimise both the environmental variable set and beta multiplier for our MaxEnt model. We tested beta multipliers between 1 and 15 using a 0.5 step change and predictors variables were chosen using a contribution threshold of 5% and a correlation threshold of 0.9. The optimised model under these parameters included four predictors (Bio04 temperature seasonality, Bio08 mean temperature of wettest quarter, Bio19 precipitation of coldest quarter and elevation) and a beta multiplier of 1 and was run in the standalone implementation of MaxEnt to produce the final model presented here.

## Results

### Data overview

Reads per sample generated by DArTseq ranged from 1,598,695 to 5,256,113, excluding the failed *E*. *lucens* sample (MEL0278390) which returned only 54,343 reads. A total of 43,054 loci were reconstructed by *ipyrad*, which created a total data alignment of 2,811,766 bases (79.6% missing data) used for *RAxML* ML analysis and a matrix of 108,777 SNPs (72.1% missing data) used for MP analysis. The filtered DArTseq SNP dataset (one SNP per locus) used in the STRUCTURE analysis contained 4323 SNPs (6.7% missing data).

### Phylogeny

**Fig 2:**
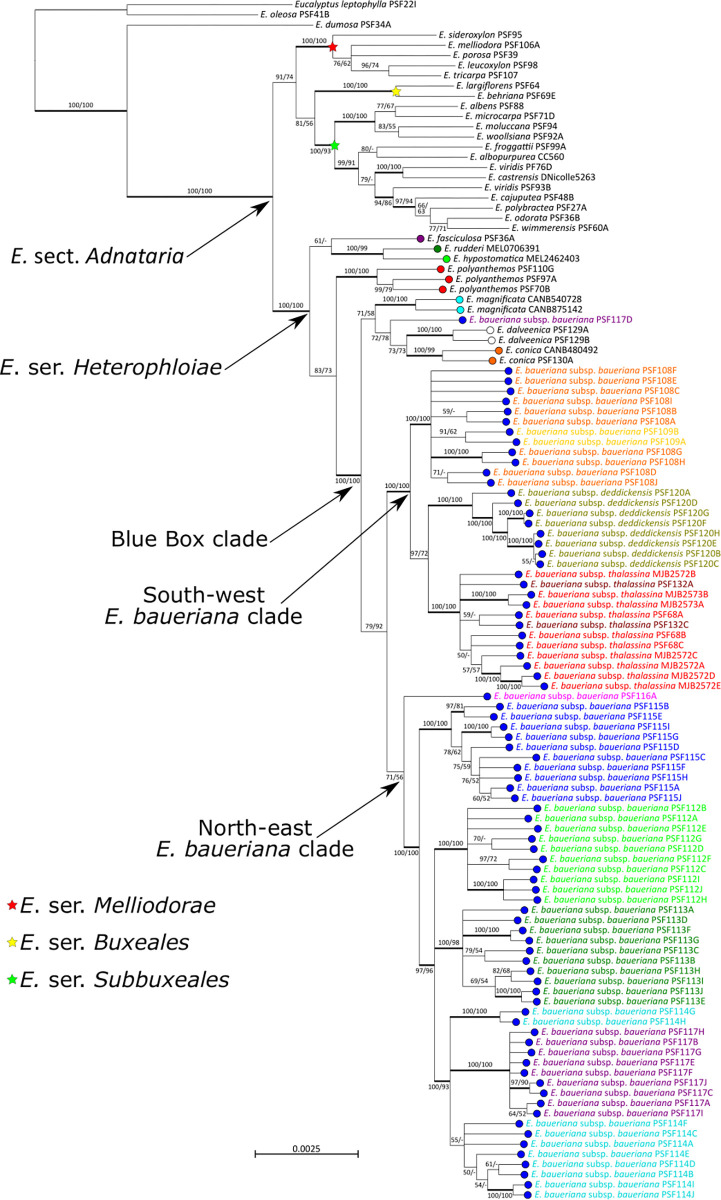
Maximum likelihood phylogeny including all samples of *Eucalyptus* sect. *Adnataria* included in this study. The phylogeny is rooted on two samples from *E*. sect. *Bisectae* and support values shown are ML and MP bootstrap values and branches with bootstrap values above 80 for both analyses are thickened. Members of *E*. ser. *Heterophloiae* are indicated by tip bubbles coloured to correspond to Fig 1A and tip labels of *E*. *baueriana* are coloured by their collecting site of origin matching Fig 1B.

Although there were some topological differences between the ML and MP phylogenies, there were no supported conflicts between the two analyses. The resulting phylogeny (Fig 2) shows that *E*. series *Heterophloiae* is the monophyletic sister group to the rest of our section *Adnataria* samples from *E*. series’ *Melliodorae*, *Buxeales* and *Subbuxeales*, though the later clade is not supported at an 80% bootstrap threshold in the MP analysis (Fig 2). Two clades form at the base of the series; an unsupported one consisting of the *E*. *fasciculosa* sample sister to *E*. *rudderi* and *E*. *hypostomatica*, and a clade only supported in the ML analysis containing *E*. *polyanthemos* and the Blue Boxes including *E*. *baueriana*. The three samples of *E*. *polyanthemos* formed a supported clade, with the Gippsland sample sister to the two samples from central Victoria. The Blue Box species, *E*. *baueriana*, *E*. *conica*, *E*. *magnificata* and *E*. *dalveenica*, form a supported clade sister to *E*. *polyanthemos*, and apart from a single sample of *E*. *baueriana* from the Ropes Creek site (PSF117D), these species were each supported as monophyletic in the MP analysis, although in the ML analysis support for the *E*. *baueriana* clade falls just short of the 80% bootstrap threshold. Relationships between these Blue Box species are less supported, with the topology suggesting *E*. *baueriana* is sister to the other three taxa, with *E*. *conica* and *E*. *dalveenica* being sister and the *E*. *baueriana* sample PSF117D and *E*. *magnificata* being sister to these species in turn. The ABBA-BABA test (D-statistic = 0.223, D-statistic bootstrapped standard deviation = 0.089, Z-score = 2.500) showed bias in shared derived alleles between the *E*. *baueriana* sample in this clade and *E*. *conica* compared to the remaining samples collected at Ropes Creek but did not reach the commonly used significance threshold of a Z-score > 3 [50].

Within the *E*. *baueriana* clade two geographic clades form, one of which is supported in both ML and MP analyses and which we call the south-western clade contains samples from the Boggy Creek and Tambo River sites from the eastern Gippsland Plain/western East Gippsland Lowlands region, the Deddick River site, and the two sites in the Werribee River catchment at Long Forest and Werribee Gorge (Fig 2). The second, which lacks support in both analyses, contains all samples from Maramingo Creek in the Genoa River/Wallagaraugh River catchment at the eastern side of the East Gippsland Lowlands, and all sites to the north of this in NSW excluding the Ropes Creek sample PSF117D, and which we call the north-eastern clade. Within the south-western clade, three main supported clades are present corresponding to the three catchments sampled from, with the ML but not the MP analysis supporting the Deddick River and Werribee River catchments as sister clades. Few relationships are supported within these clades; however, several samples in the Deddick River clade are likely closely related to one another, possibly even siblings, given their short tip branches.

The sole Redbank Creek sample is sister to the rest of the north-eastern clade. The remainder of the north-eastern clade is supported as a single lineage and four of the five sampling sites form supported clades, with only the Tuross River plants not being monophyletic. The Bendalong samples were supported as the sister lineage to the four other sites, which is unexpected given the location is between the Ropes Creek site on the Cumberland Plain and the three other sites to the south. Relationships between the four remaining sites are not resolved with the Maramingo Creek clade, Doctor George Mountain clade and a clade containing the Tuross River and Ropes Creek samples. Within the Tuross River and Ropes Creek clade, the Ropes Creek samples show very limited genetic divergence from one another and form a supported clade that is part of a polytomy with the Tuross River samples between which relationships are largely unresolved.

### *Eucalyptus baueriana* population genetics and structure analysis

*F*_st_ and heterozygosity values for *E*. *baueriana* populations (Table 3) show lower observed heterozygosity than expected heterozygosity and a high overall *F*_is_ (0.286), providing evidence for inbreeding at most collection sites. The overall *F*_st_ value (0.218) for the species is high, indicating strong genetic structuring of the species, which reinforces the lack of geneflow between populations. Two isolated populations, Deddick River and Ropes Creek, show consistently high *F*_st_ values when compared to all other populations. Of the remaining populations, those that formed the south-western clade in the phylogeny (discussed below), bar the Deddick River, shared lower reciprocal *F*_st_ values, which is also true of all sites from the NSW south coast and Maramingo Creek in far eastern Victoria that are members of the north-eastern clade in the phylogeny.

**Table 3:** Population statistics for the ten sampling sites of *E*. *baueriana* investigated in this study. Cells showing interpopulation *F*_st_ values are coloured by the magnitude of the value, with redder cells having higher *F*_st_ values and bluer cells having lower.

Population	Number of individuals	Population H_o_	Population H_e_	Population *F*_is_ value	Interpopulation *F*_st_ values								
					Deddick River	Werribee River Gorge	Pyrites and Djerriwarrh Creeks	Tambo River	Boggy Creek	Maramingo Creek	Doctor George Mountain	Tuross River	Bendalong
Deddick River	8	0.0929	0.0965	0.0376									
Werribee River Gorge	2	0.0920	0.0864	-0.0647	0.3665								
Pyrites and Djerriwarrh Creeks	10	0.0942	0.1352	0.3031	0.3001	0.0126							
Tambo River	10	0.1091	0.1490	0.2679	0.2860	0.1011	0.1168						
Boggy Creek	2	0.0981	0.0939	-0.0439	0.3964	0.1365	0.1478	0.0422					
Maramingo Creek	10	0.1139	0.1535	0.2580	0.3344	0.1886	0.2134	0.1486	0.1607				
Doctor George Mountain	10	0.1050	0.1394	0.2472	0.3633	0.2300	0.2407	0.1644	0.1963	0.0850			
Tuross River	10	0.1051	0.1427	0.2632	0.3562	0.2174	0.2311	0.1676	0.1886	0.0877	0.0896		
Bendalong	10	0.1022	0.1357	0.2469	0.3804	0.2626	0.2616	0.1948	0.2294	0.1342	0.1455	0.1227	
Ropes Creek	9	0.1019	0.0931	-0.0940	0.5058	0.4418	0.3736	0.3151	0.4229	0.2454	0.2433	0.2148	0.2803

**Fig 3:**
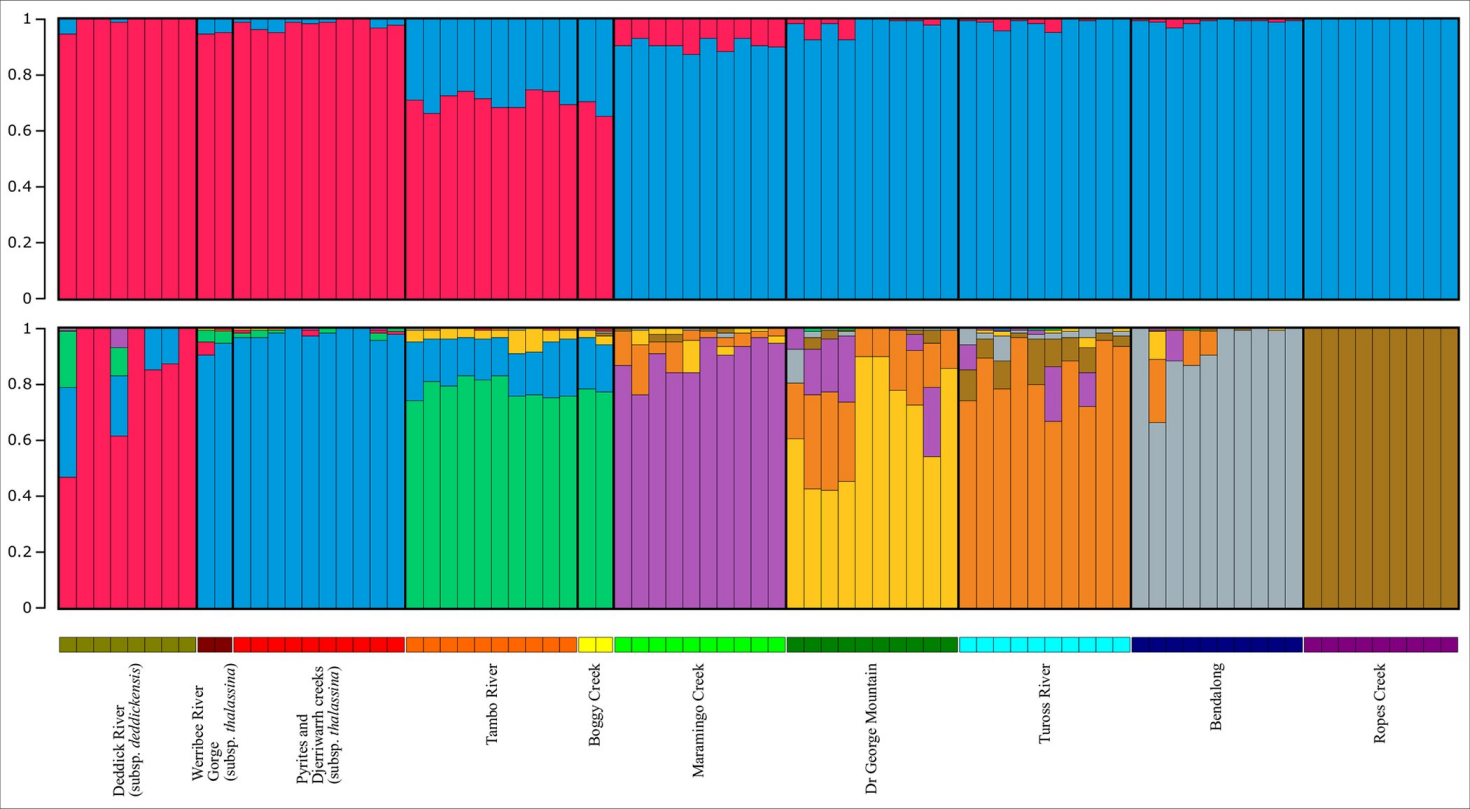
Structure plots showing clustering of *E*. *baueriana* samples at the two optimal values of K: K = 2 (top) which correspond to the two main clades found in the phylogeny (Fig 2) and K = 8 (bottom) where clusters correspond to the eight major areas sampled.

Two K values showed high delta K and likelihood scores, two and eight. While delta K skews towards favouring K = 2 [51], this value of K is still presented here as it fits with our a priori knowledge of the two major clades in the phylogeny, while K = 8 matches the eight main sampling regions included in the dataset. Indeed, when the results for these two K-values are plotted these expectations are realised. As shown in Fig 3, the two clusters in the K = 2 analysis correspond broadly to the two clades in the phylogeny, a south-eastern cluster containing the Werribee River catchment (Werribee River Gorge and Pyrites and Djerriwarrh creeks samples), Deddick River, and Tambo River samples. The Tambo River samples are suggested to share greater ancestry with the north-eastern lineage than the first two. Reciprocally, we see the highest share of south-western ancestry of any north-eastern population in samples from Maramingo Creek, the site nearest the south-western populations.

In the K = 8 analysis, the 8 ancestry’s largely correspond to the eight main regions our samples are sourced from; the Werribee River catchment (including both the Werribee River Gorge and Pyrites and Djerriwarrh creeks sampling sites), the Deddick River, the eastern Gippsland Plain/western East Gippsland Lowlands (including both the Tambo River and Boggy Creek sites), Maramingo Creek (Genoa River/ Wallagaraugh River catchment), Doctor George Mountain (Bega River catchment), the Tuross River, Bendalong, and Ropes Creek (Cumberland Plain). The ancestry that corresponds to the Deddick River samples is largely absent from other samples, however there is a significant contribution of the Werribee River catchment lineage and the eastern Gippsland Plain/western East Gippsland Lowlands lineage into the Deddick River samples. There is also a small reciprocal contribution between samples in these two other lineages as well, however, in this analysis, there is very limited sharing of ancestry between the south-eastern and north-western clades. There was less complete separation of samples from the Maramingo Creek, Doctor George Mountain and Tuross River into distinct lineages, with contributions from each of the other’s ancestry being present in most samples, along with small contributions from the Bendalong and Ropes Creek lineages. The Bendalong samples mainly formed a single lineage but had small contributions from the Tuross River and Doctor George Mountain lineages. The Ropes Creek samples formed their own distinctive lineage.

### Species distribution models

**Fig 4:**
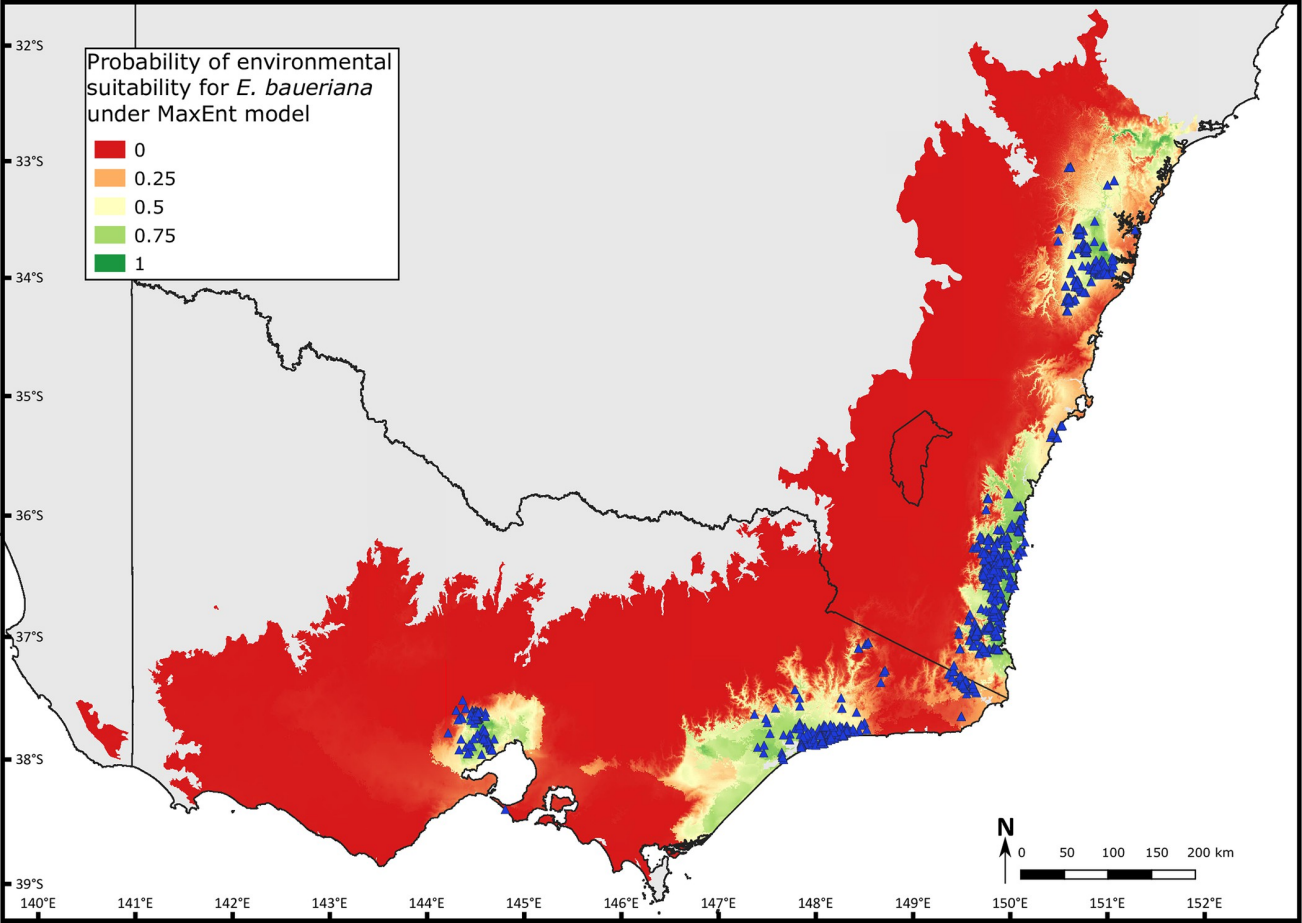
Map showing predicted environmental suitability in south-east Australia for *E*. *baueriana* based on MaxEnt model. Four environmental predictor variables were employed in the model: Bio04 temperature seasonality, Bio08 mean temperature of wettest quarter, Bio19 precipitation of coldest quarter and elevation. Greener areas show higher predicted suitability while redder areas have lower predicted suitability, with blue triangles representing records of *E*. *baueriana* downloaded from Atlas of Living Australia (accessed 30 November 2020) and used to build model. The base map is adapted from [14] under a CC BY 3.0 AU license.

As shown in Fig 4, with only the four predictor variables MaxEnt returned a modelled suitability that strongly reflects the known distribution of the species. We see breaks in environmental suitability corresponding to three disjunctions, the Strzelecki Ranges and western Gippsland Plain, the Gippsland Lowlands, and the Illawarra district, however we also see areas of high suitability outside where the species is known to occur. There is a large extension of suitable habitat extending across a much greater portion of the eastern half of the Gippsland Plain than the known range of the species, no break in suitability corresponding to the disjunction between the south-east coast and Bendalong populations, and a large area of higher suitability north of the Cumberland Plain.

Bio08 (mean temperature of the wettest quarter) was the most important variable in the model with the modelled response to this variable suggesting that the species favours areas which are not winter rainfall dominated (Table 4). The inclusion of Bio04 (temperature seasonality) and Bio19 (precipitation of coldest quarter) in the optimised model (Table 4) corroborates this hypothesis that seasonality of temperature and rainfall are more important in determining the species distribution than annual averages. Elevation was the second most important predictor (Table 4), which is not unexpected given the species is mostly found on coastal plains and lowlands but is a complex variable which correlates with many environmental factors that may affect the distribution of *E*. *baueriana* more directly.

**Table 4:** Contribution and importance of variables in the optimised MaxEnt model for *E*. *baueriana*.

Variable	Percent contribution	Permutation importance
Temperature—wettest quarter mean (Bio08)	46.4	43.6
Elevation	31.1	24.2
Temperature Seasonality (Bio04)	15.7	19.4
Precipitation of Coldest Quarter (Bio19)	6.8	12.8

## Discussion

### Phylogeny of *E*. ser. *Heterophloiae* and placement of *E*. *baueriana*

Our results support the monophyly of *E*. series *Heterophloiae* as currently defined, although we could not get data from our *E*. *lucens* sample to confirm its placement in the series. The subsectional classification of Brooker [52], which places series *Heterophloiae* together with series *Melliodorae* in subsection *Terminales*, is not supported (Fig 2). Rather, we find that series *Melliodorae* is grouped with members of subsection *Apicales* (series *Buxeales* and *Subbuxeales*) in the phylogeny (Fig 2).

Within *E*. series *Heterophloiae* inferred relationships between taxa are only somewhat congruent with what has been hypothesised in previous classification schemes. The relationship between *E*. *fasciculosa* and other members of the series was unclear, and the single sample of this species formed part of a polytomy at the crown of *E*. series *Heterophloiae* along with the *E*. *rudderi*/*E*. *hypostomatica* and *E*. *polyanthemos*/Blue Box clades. We find *E*. *polyanthemos* to be sister to the Blue Box clade, which fits with the thoughts of previous authors [11, 16, 53]. While Collins et al. [16] considered *E*. *conica* to be a member of the Blue Box group with *E*. *baueriana*, *E*. *magnificata* and *E*. *dalveenica* matching our phylogeny, both Brooker [52] and Nicolle [11] considered *E*. *hypostomatica*, *E*. *rudderi* and *E*. *conica* to form a group. Our results suggest that *E*. *baueriana* is the sister lineage to the other three species of Blue Box, *E*. *conica*, *E*. *dalveenica* and *E*. *magnificata*, with the former two possibly being sister taxa. However, the bootstrap support values on several key branches do not reach the 80% support threshold in one or both analyses, and a sample of *E*. *baueriana* from the northernmost sampled population at Ropes Creek falls in the clade containing the three other Blue Box taxa.

Collins et al. [14] identified stable populations of putative *E*. *conica × E*. *magnificata* hybrids, although work to confirm the exact identity of this entity is ongoing. This suggests hybridisation between the Blue Box species may occur where there is sympatry or parapatry. While there is no distributional overlap between *E*. *baueriana* and *E*. *conica*, the Ropes Creek site was the closest sampled to *E*. *conica* populations. We hypothesise the placement of the *E*. *baueriana* sample from this site outside the main *E*. *baueriana* clade may be due to historical hybridisation leading to introgression of *E*. *conica* genetic material into this population of *E*. *baueriana*. While our ABBA-BABA test does not show significant support for this hypothesis, there is weak evidence to reinforce it in the form of the D-statistic of 0.223 which indicates a slightly higher number of shared derived alleles between *E*. *conica* and the *E*. *baueriana* sample in question than between *E*. *conica* and other *E*. *baueriana* samples collected at Ropes Creek. Phantom hybrids have been observed in other Australian plant taxa, where there is a genetic signal for introgression from a species which does not currently co-occur with the introgressed population [54], including in eucalypts [55–57], Additionally, this introgressed sample can be isolated as the cause of the low bootstrap values on the branches between the Blue Box species, as subsequent phylogenetic analyses with this sample removed from the dataset resulted in supported relationships placing *E*. *conica* and *E*. *dalveenica* as sister taxa, with *E*. *magnificata* sister to this clade (S1 Fig).

#### Subspecific classification

Despite the two geographically restricted subspecies of *E*. *baueriana* being supported as sister clades in our phylogeny, they are nested within *E*. *baueriana* subsp. *baueriana*. The eastern Gippsland Plain/western East Gippsland Lowlands populations of the nominotypical subspecies are supported as more closely related to the two isolated subspecies than to other populations of *E*. *baueriana* subsp. *baueriana*. This suggests that if subspecies that represent distinct and monophyletic lineages are desired the current infraspecific circumscription needs to be reassessed. While it may be the case that monophyletic subspecies are not desired, nor necessarily expected given the divergence of a monophyletic peripheral population can occur without a dichotomous split with the remaining populations of a taxon which are rendered paraphyletic [58, 59], we support the approach of previous authors to synonymise all subspecies [10, 11]. While the subspecies of *E*. *baueriana* were erected using an implied morphological subspecies concept, the overlapping and poorly defined morphological character states used to distinguish the existing subspecies (plant stature, leaf size and colour, bud size and pedicel length, and fruit size) [10, 19] makes identifying the subspecies on any basis other than geography difficult. This lack of morphological differentiation is only reinforced by our findings that there is no greater genetic divergence between the three subspecies in the south-western clade than the level of genetic differentiation amongst populations of *E*. *baueriana* subsp. *baueriana* which occurs in both the south-western and north-eastern clades.

If we wished to maintain an infraspecific classification scheme we could accept that the nominotypical subspecies is not monophyletic and maintain the current classification, which is often considered a valid approach at the infraspecific level [60]. However, if we wish to apply a phylogenetic subspecies concept that emphasises monophyly, further work could be undertaken to determine if there are morphological differences between the Gippsland populations and other populations of *E*. *baueriana* subsp. *baueriana* that form the north-eastern clade in our phylogeny. If consistent morphological differences can be identified, either a new subspecies could be erected to include the Gippsland populations, or these populations could be united with the other two subspecies in the south-western clade. The latter would result in a classification scheme that reflects the two genetic clades we found in our analyses, with a disjunction between them across the central Gippsland Lowlands. A further option not explored here, but given preliminary support by previous findings of seedling leaf morphology differences between populations on the Cumberland Plain and at Bendalong [16], is an infraspecific classification scheme more in line with the result of our K = 8 STRUCTURE analysis with different taxa occurring in each major catchment area.

### Phylogeography of E. baueriana

We report the highest *F*_st_ value (0.218) for a *Eucalyptus* species we are aware of to date (0.04 in *E*. *melliodora* A.Cunn. ex Schauer [61], 0.017–0.018 in *E*. *albens* Benth. and *E*. *sideroxylon* A.Cunn. ex Woolls [62], 0.066 in *E*. *behriana* F.Muell. [25], *E*. *globulus* Labill. *sensu lato* = 0.08 [63]), and despite wide acceptance of a strong inbreeding depression in the eucalypts [64], we find evidence of high inbreeding in *E*. *baueriana*. This indicates that there is stronger geographic structuring of the genetic diversity of the species and more limited gene flow between populations than in previously studied species. This is corroborated by the strong support for relationships between sampling sites in the phylogeny, despite previous studies using similar data failing to resolve interspecific relationships in *E*. sect. *Adnataria* [65]. There are multiple potential variables that could lead to this pattern, although there are two main ones that are relevant here and it is likely both have played a role in the evolution of the current pattern of genetic diversity: firstly, that the isolation and restriction of geneflow between populations of the species is comparatively old, and secondly that the ecology of the species and the nature of its populations are playing a role. The species occurs as localised, restricted populations in specific river catchments and, being closely associated with waterways, the populations are relatively linear and constrained in their occupancy of the landscape; both factors likely limit the potential for geneflow between populations.

The largest area of occurrence of *E*. *baueriana* on the south coast of NSW is within the region included in the STZ by previous authors [2, 4], and we find the basal divergence of lineages of the species is near this region, corresponding to the disjunction between populations in the east and west of the East Gippsland Lowlands. This finding is unexpected, as this disjunction is across a significantly smaller distance and more similar ecosystems than other disjunctions in the species range, especially that between the Werribee River Catchment populations and the eastern Gippsland Plains/western East Gippsland Lowlands populations.

This Gippsland disjunction corresponds to the area of higher rainfall in the central area of the East Gippsland Lowlands. Given that *E*. *baueriana* most commonly occurs in riparian areas in open forest and woodland communities associated with drier sites [10], this may indicate the species is outcompeted by other species in the area that are better suited to the closed rainforest habitat associated with the higher rainfall. This is reinforced by the findings of White et al. [66], who showed that in Victoria, gallery rainforest is most widespread in riparian habitats in the central parts of the East Gippsland Lowlands, roughly corresponding to the disjunction in *E*. *baueriana’s* distribution. The results of our SDMs hint at the relationship with rainfall being less about absolute annual rainfall, rather the amount of winter rainfall received as the areas *E*. *baueriana* occurs have limited seasonality to summer dominated rainfall, and the two southern disjunctions correspond to areas with more winter dominated rainfall. We also see evidence that the species distribution may be limited by how cold winters get on average.

However, we must make sure to distinguish between an environmental condition maintaining a disjunction and a possible vicariance event. Long distance colonisation is unlikely in eucalypts given their lack of seed dispersal mechanisms [67] and has been assumed to not be a major factor in other eucalypt species with disjunct distributions [25, 68, 69]. This leads to the hypothesis that the populations in the eastern and western East Gippsland Lowlands must have had a more or less continuous area of occurrence at some point in the past and vicariance is responsible for the current disjunction between them.

The most broadscale cause of recent natural vegetation change in south-east Australia were the climatic shifts between glacial and interglacial climates during the Quaternary, with glacial periods likely resulting in lower temperatures and decreased rainfall [70, 71], although some environmental records support there being higher rainfall in at least some parts of south-east Australia under glacial climates [72]. If it is high rainfall maintaining the disjunction in the species distribution, it can be hypothesised lower rainfall under glacial climates may have allowed the species to be more widespread, with continuous population from the Sydney Basin to the Werribee River catchment, or some points in between given we cannot rule out northward or westward expansion of the species since then due to the limited genetic distinction of these peripheral populations.

Our data suggest that isolation of the Werribee River Catchment populations is relatively recent given their close genetic relationship to those on the Deddick River. Previous findings of Fahey et al. [25] showed that the isolation of a population of another eucalypt species, *E*. *behriana*, at Long Forest in the Werribee River Catchment was one of the most recent vicariance events across the distribution of that species which otherwise occurs inland of the Great Dividing Range. While *E*. *behriana* occurs in very different environments to *E*. *baueriana*, together these patterns suggest that there may have been major changes of vegetation patterns in this part of Victoria in the recent past, possibly tied into glacial-interglacial climate fluctuations or volcanic activity [21, 25].

Our data do little to explain the presence of the populations on the Deddick River, other than providing strong evidence they do represent outlying populations of *E*. *baueriana*. Being most closely related to the populations in the Werribee River catchment is an unexpected result, given the substantial geographic separation and the presence of intervening populations in the Gippsland Plain and East Gippsland Lowlands, but may fit with the descriptions of these two subspecies sharing some morphological traits [19]. The geographic changes that may have led to this relatedness are less clear. While we see limited genetic divergence in many of the eight samples from the Deddick River in our phylogeny (Fig 2), this largely reflects our restricted sampling. We sampled from the smaller of the two populations known at the time of publication of the subspecies, representing only 14 individuals of the estimated total 200 individuals on the river, as the steep terrain along the Deddick River makes accessing the river banks difficult, likely reflecting why the populations weren’t represented by collections prior to 2005 [19].

In the north-eastern clade, the population structuring appears to predate the isolation of the populations on the Cumberland Plain, at Bendalong, and the far south coast. Both the seedling morphology and genetic data examined by Collins et al. [16] suggested a level of divergence between populations on the Cumberland Plain and at Bendalong, which our data corroborate, with the Bendalong population diverging earlier than the populations on the Cumberland Plain and the far south-coast. This is despite the Bendalong population being the most central, siting nearly halfway between the other two. It seems unlikely that there was geographic connectivity between the Cumberland Plain and the far south coast populations post isolation of the Bendalong population as the major inland barrier between these populations is the higher elevations to the north and west of the Illawarra district, a barrier not likely to have been permeable in the past, and therefore historical connectivity was likely along the coast which would have included the Bendalong area. Due to this, we find ourselves unable to put forward any solid biogeographical hypotheses to explain why the Bendalong population diverges early within the north-eastern clade. It is possible that the cause is not biogeographical, but rather the result of genetic processes such as incomplete lineage sorting between the populations, founder effects or greater genetic drift due to the small population size at Bendalong.

The isolation of these three areas of occurrence (Cumberland Plain, Bendalong and the South Coast) might be maintained by the higher elevation of the Illawarra escarpment and higher rainfall in the intervening areas. There is a narrower coastal plain in the Illawarra district than further south on the NSW coast, limiting suitable habitat for *E*. *baueriana*, and the higher rainfall produces more closed forests. While the coastal shelf off eastern Australia is narrow (~20 km to ~45 km within the study region) [70, 73], under the modal sea levels of glacial climates, including the last glacial maximum (LGM), there would have been a slightly wider coastal plain [74]. Under a hypothesised lower rainfall regime [70] this may have benefitted *E*. *baueriana*, providing more flat land habitat and leading to more continuous occupancy of the south-east corner of the Australian mainland than at present. This wider, more continuous population could have then undergone vicariance due to the narrowing of the coastal plain and expansion of closed forests as rainfall and sea level increased moving into an interglacial period.

### Conclusions

In this study we have shown that there is strong genetic structuring corresponding to the disjunct populations of *E*. *baueriana*, with levels of inbreeding that are high for a eucalypt species, potentially playing a role in establishing this structure. The earliest divergence between populations of the species is in the Gippsland Lowlands, forming two genetic groupings corresponding to south-western population and north-eastern populations. These two main genetic groups do not correspond to the three described subspecies of *E*. *baueriana*, as the nominotypical subspecies is represented in both groups while the two geographically restricted subspecies forming part of the south-western clade. Additionally, we have shown the Blue Box group, consisting of *E*. *baueriana*, *E*. *conica*, *E*. *magnificata* and *E*. *dalveenica*, represents a monophyletic grouping with *E*. ser. *Heterophloiae* most closely related to *E*. *polyanthemos*.

## Supporting information

S1 Fig Maximum likelihood phylogeny excluding *E*. *baueriana* sample that shows evidence for introgression from *E*. *conica* rooted on two samples from *E*. sect. *Bisectae*. Support values shown are ML and MP bootstrap values and branches with bootstrap values above 80 for both analyses are thickened. Members of *E*. ser. *Heterophloiae* are indicated by tip bubbles coloured to correspond to Fig 1A and tip labels of *E*. *baueriana* are coloured by their collecting site of origin per Fig 1B.
(TIF)
