# Supplementary figures and images for: A box on the river: The phylogenetics and phylogeography of Eucalyptus baueriana (Eucalyptus sect. Adnataria ser. Heterophloiae)
Source: PLoS One. 2022 Nov 17;17(11):e0276117. doi: 10.1371/journal.pone.0276117 (PMC9671351; doi:10.1371/journal.pone.0276117)

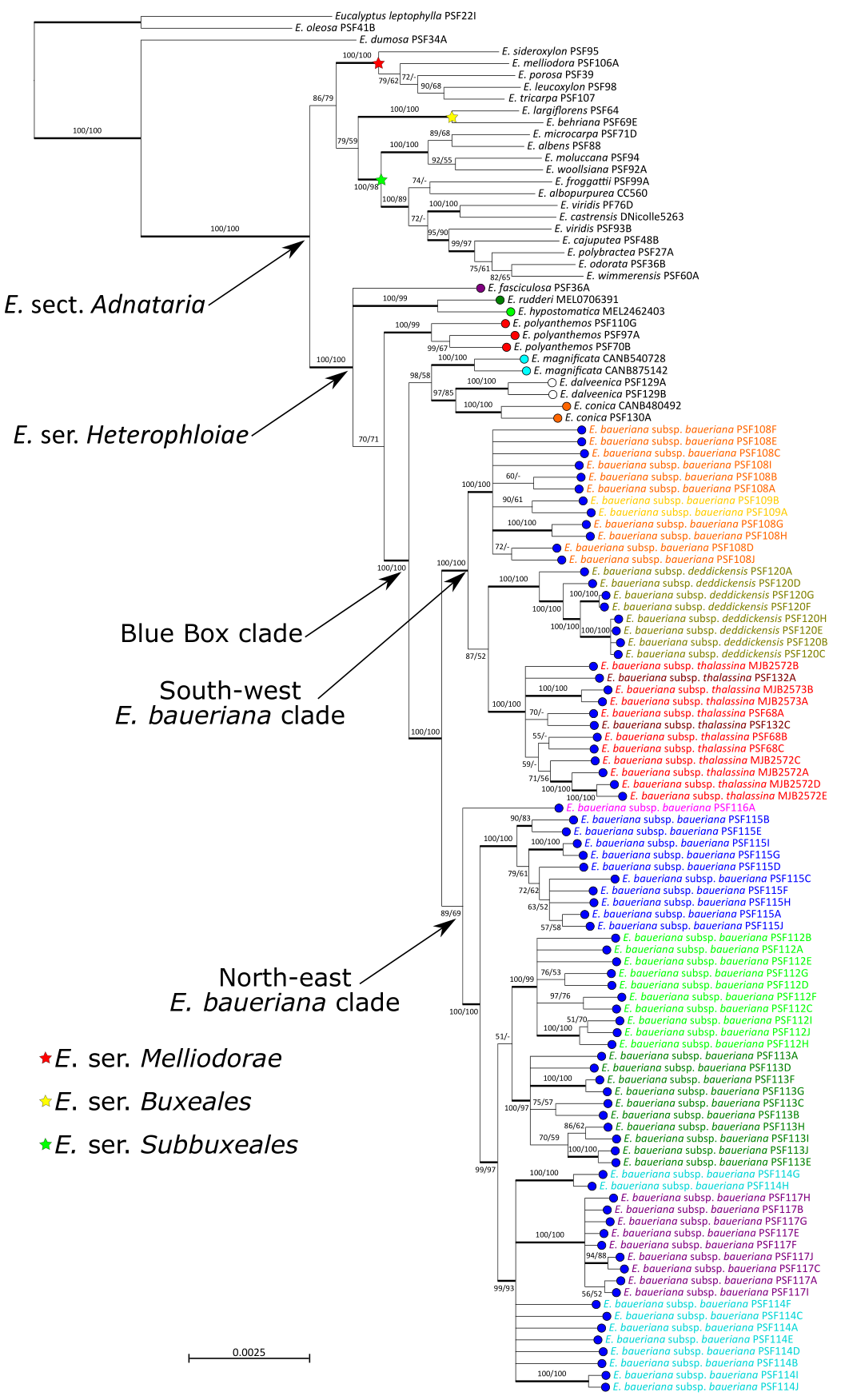

Supplement: S1 Fig — Support values shown are ML and MP bootstrap values and branches with bootstrap values above 80 for both analyses are thickened. Members of E. ser. Heterophloiae are indicated by tip bubbles coloured to correspond to Fig 1A and tip labels of E. baueriana are coloured by their collecting site of origin per Fig 1B. (TIF) [file pone.0276117.s001.tif]
